# Help-seeking behavior in bereaved university and college students: Associations with grief, mental health distress, and personal growth

**DOI:** 10.3389/fpsyg.2022.963839

**Published:** 2022-08-03

**Authors:** Emilie Tureluren, Laurence Claes, Karl Andriessen

**Affiliations:** ^1^Faculty of Psychology and Educational Sciences, KU Leuven, Leuven, Belgium; ^2^Faculty of Medicine and Health Sciences, University Antwerp, Antwerp, Belgium; ^3^Centre for Mental Health, Melbourne School of Population and Global Health, The University of Melbourne, Parkville, VIC, Australia

**Keywords:** social support, bereavement, students, help-seeking, personal growth, mental health, grief

## Abstract

Many students have experienced the death of a loved one, which increases their risk of grief and mental health problems. Formal and social support can contribute to better coping skills and personal growth in bereaved students. The purpose of this study was to examine the support that students received or wanted to receive and its relation to students’ mental health. We also looked at students’ needs when receiving support and barriers in seeking formal and social support. Participants (*N* = 666) completed an online survey consisting of questions about their sociodemographic characteristics, the support they received or wanted to receive, and support needs and barriers in seeking support. The survey also included three scales assessing grief, mental health distress, and personal growth. First, we analyzed the data descriptively. Next, we used MANCOVA to examine whether students who did or did not receive or wanted more support differed in terms of their grief, mental health distress, or personal growth. About 30% of students needed more support and experienced more grief and mental health distress than students who had their support needs met. Students who received support experienced more personal growth and grief than students who did not receive support. Students indicated a need for feeling acknowledged and safe. Feeling like a burden to others and perceiving their problems as not serious enough to warrant support were common barriers to seeking support. Our results indicate that support should be provided actively to students after the death of a loved one, and support being available on an ongoing basis.

## Introduction

Experiencing the death of a close person, such as a family member or a friend, is a disruptive and one of the most distressing events in the lives of students, with a potentially lasting impact (Balk et al., 2010). Between 22 and 30% of students have experienced the death of a close person in the past year (Balk, 2008). Common acute grief reactions include feelings of sadness, anger, and guilt. In addition, bereaved students can experience sleeplessness, loss of energy, and decreased motivation which, in turn, may affect their academic performance (Cupit et al., 2016; Elsner et al., 2022). Students are also vulnerable for developing mental health problems, such as anxiety, mood, and substance abuse disorders, and problems with social functioning (Alonso et al., 2018; Auerbach et al., 2018). Experiencing bereavement can exacerbate mental health problems in students, for example, regarding posttraumatic stress disorder or prolonged grief disorder, especially after sudden and traumatic deaths such as suicide (Fowler, 2006; Cooley et al., 2010).

Eilertsen et al. (2013) reported that study participants who received little or no support after the death of their sibling had a greater probability of experiencing more anxiety than siblings who had their support needs met. Social support provides the opportunity for self-disclosure, which allows processing negative feelings, and creates mental space to engage in meaning-making after the death, which, in turn, facilitates personal or posttraumatic growth (Cohen and Samp, 2018). Personal growth is the result of a person going through a challenging life event, such as the death of a loved one. This life event causes the person to reflect on their life and try to find meaning after it (Hogan and Schmidt, 2002; Taubman Ben Ari et al., 2011). Participants in a study by Rosenberg et al. (2015) expressed that as a result of the death of their loved one, they became more mature and experienced more kindness for themselves and others. Posttraumatic growth refers to a similar phenomenon, but it differs from personal growth in that the life event needs to be traumatic for the person to experience growth (Feigelman et al., 2009; Taubman Ben Ari et al., 2011).

Reaching out to peers who have had similar experiences, or support groups, may provide emotional support and decrease feelings of loneliness in students (Pitman et al., 2018; Andriessen et al., 2019; Azorina et al., 2019). However, other bereaved students may have doubts about joining a support group because of the confrontation with their and others’ grief reactions. Students can also seek information and help through online resources (Hao et al., 2016). Self-help interventions and online therapy have grown in popularity in recent years; however, while most resources provide information, evidence-based online bereavement support is still scarce (Beaunoyer et al., 2020).

Andriessen et al. (2019) reported that only a few of their participants had found their way to formal help on their own initiative after the death of a loved one. Therapy helped some participants to gain insight into the grieving process, their feelings of guilt, and/or the context of the death. Negative experiences during therapy sessions caused participants to discontinue help (Andriessen et al., 2019). Participants in the study by Pitman et al. (2018) indicated that they needed professional support, or at least felt that they should know that this was available if they needed it. This reassured them and was experienced as taking pressure off family and friends.

Studies have suggested that students have certain needs and expectations regarding the formal and social support they receive after the death of a loved one. Participants in the study by Pitman et al. (2018) wanted to know that someone was there for them and that they were not going to be treated different than before the bereavement. For some, this meant friends acknowledging their loss and giving them the opportunity to talk about it. Others did not want to talk about the death and wanted to be treated as before the bereavement (Pitman et al., 2018). Similar results were reported in the study by Andriessen et al. (2020). Emerging adults, and specifically students, indicated in several studies that they experienced barriers to seeking help such as lack of knowledge of available help, cultural stigma, feelings of uneasiness, being a burden or being judged, and feelings of guilt (Pitman et al., 2018; Andriessen et al., 2019; Azorina et al., 2019; Tan and Andriessen, 2021). Mistrust in certain services, possibly based on previous experiences, specifically hindered seeking formal help as those bereaved feared being treated badly or not feeling accepted. Negative experiences with professionals reinforced the feeling that other people were not interested or cannot understand them (Pitman et al., 2018; Andriessen et al., 2019).

Students are going through a developmentally vulnerable stage of life during which they face challenges, such as learning to live independently and moving away from their original support system such as family and friends (Arnett, 2000). Also, students’ culture is often “fun” oriented including easy access to alcohol or other substances, which may further hinder receiving adequate support after the death of a loved one (Osberg et al., 2010). Given the potentially lasting ramifications of bereavement on students’ lives, it is important to understand the support needs of these students so mental health professionals and the bereaved students’ social support system can support them in coping with their loss. Nonetheless, few studies have examined help-seeking in bereaved university and college students, few studies have explored students’ support needs and barriers to support, and some studies included younger age groups as well. Furthermore, as local contexts can affect help-seeking, nothing is known about help-seeking of bereaved university and college students in Belgium, and how their experiences compare to those reported in other studies. By knowing and understanding what their support needs are and being able to offer suitable support as soon as possible, grief and mental health deterioration, and subsequent needs for more intensive care can be prevented. It can help students who feel stuck in their grieving process and contribute to experiencing personal of posttraumatic growth (Tedeschi and Calhoun, 2004).

In this study, we aimed to investigate (1) the support that bereaved students received and the support that they wished they had received and (2) its relation to grief, mental health distress, and personal growth. We also explored (3) what students find important when receiving support (e.g., feeling safe), as well as (4) which barriers students experience when seeking formal and social support.

## Materials and methods

### Study design and sampling

Eligible participants had to (1) be enrolled in a Flemish university or college, (2) be between 18 and 28 years old, (3) have experienced the death of a loved one after their 12th birthday (to prevent recall bias), and (4) the death had occurred at least 6 months before participating in the study. We disseminated the study announcement *via* approximately 200 student organizations, and *via* social media (Twitter, Facebook, and LinkedIn). Participants could enroll in a draw to win one of five 20.00 Euro gift vouchers. The Social and Societal Ethics Committee of the KU Leuven—University of Leuven approved the study (G-2019 101,762, March 19, 2020).

Using G*Power 3.1.9.7 (Faul et al., 2009), we determined the minimum sample size for a MANCOVA at *N* = 270. A total of 1,390 people started the survey. Of these, 488 people were excluded based on the inclusion criteria, and another 236 people were excluded due to missing data. The final sample included 666 participants (*M*_age_ = 21.42, *SD* = 1.97, range 18–28 years).

### Measures

The study was part of a larger research project on grief and mental health in students, which implies that the survey included more topics than discussed here. We created an online survey in Qualtrics to collect the data. The survey started with sociodemographic questions about participants’ age, gender, and relationship to the deceased person. We translated two standardized questionnaires following the steps recommended by the World Health Organization (WHO, n.d.): AGI (Andriessen et al., 2018) and HGRC-PG (Hogan et al., 2001). We also used the Dutch version of the DASS-21 (de Beurs et al., 2001). In the absence of an existing instrument suitable for this study, the questions about formal and social support, student’s support needs, and barriers to seeking support were created for this study based on the literature (e.g., Pitman et al., 2018; Andriessen et al., 2019). The design of the online survey was tested by three people (who met the inclusion criteria). They gave feedback on the clarity of the questions and instructions. In addition, it was tested whether the questions were answered correctly and how long it took to complete the survey (i.e., an average of 15 min). The online survey was accessible between March and October 2020.

#### Grief

The Adolescent Grief Inventory (AGI; Andriessen et al., 2018) consists of 40 items and 6 factors: sadness, self-blame, anxiety and self-harm, shock, anger and betrayal, and sense of peace (e.g., I was grateful that he/she was no longer suffering). Participants rate the items on a 5-point Likert-type scale ranging from 1 = Not at all, to 5 = Extremely. Scores are averaged; higher scores indicate more grief reactions (range 1–5). The Cronbach’s alpha in our sample was 0.89.

#### Mental health distress

The Depression Anxiety Stress Scale 21 (DASS-21, Dutch version; de Beurs et al., 2001) consists of 21 items and 3 subscales: depression, anxiety, and stress (e.g., I tended to over-react to situations). Participants reply to the statements on a 4-point Likert-type scale ranging from 0 = Never, to 3 = Almost always. Scores are summed and higher scores indicate more distress (range 0–63). The Cronbach’s alpha in our sample was 0.94.

#### Personal growth

The Personal Growth subscale of The Hogan Grief Reaction Checklist (HGRC-PG; Hogan et al., 2001) consists of 12 items (e.g., I have more compassion for others). Participants rate the items on a 5-point Likert-type scale, ranging from 1 = Does not describe me very well, to 5 = Describes me very well. Scores are summed and higher scores indicate more personal growth (range 12–60). The Cronbach’s alpha in our sample was 0.85.

#### Formal and social support

The participants were asked if they had received any formal/social support after the death of their loved one. If they answered “Yes,” they were asked from whom they had received support. They could select one or more answers from a list (e.g., friends, family, and general practitioner). By adding these three examples, participants were reminded that both social support and formal support were included in this question. Participants could also type their own answers in a free text box. All participants were then asked if they would have liked to receive more support. If they answered “Yes,” they were asked from whom they had wanted more support. They received the same list of options and could select multiple answers and use a free text box. Participants were then asked a question consisting of 6 statements about their needs in receiving support (e.g., “Help that is available when I am ready to receive help”). The participants were asked to rate on a 4-point scale ranging from “1. Not important at all” to “4. Very important” how important these statements were to them in receiving support.

Next, participants were asked if anything had held them back from seeking (social) support from friends and/or family. If they answered “Yes,” they were given a list of possible answers (e.g., “I felt like a burden”). Participants could select multiple answers and could also type their own answers in a free text box. All participants were then asked if anything had held them back from seeking formal support. When they answered “Yes,” they could answer the question in the same way as the previous question.

### Data analysis

We imported all data in SPSS 27 (IBM Corp, 2020). We used frequencies and percentages to analyze the sociodemographic data. Frequencies and percentages were also used to analyze the data regarding the received and desired support, students’ needs in receiving support, and barriers to seeking support.

We used multivariate analysis of covariance (MANCOVA) to examine whether students who (a) did or did not receive support, and (b) did or did not want (more) support differed in terms of grief, mental health distress, and personal growth. Age of participants and time since loss were included as covariates as these variables correlated with the outcome variables (grief, mental health distress, and personal growth). Because two variables (time since loss, and mental health distress) did not meet the assumption of normality of data distribution, we used a bootstrapping procedure with *n* = 1,000 resamples (Preacher and Hayes, 2008).

## Results

Most of the 666 participants identified as women (*n* = 568). The mean age of our participants was 21.42 years old (*SD* = 1.97). The mean time since the bereavement was 41 months (*SD* = 30.72). The participants scored on average *M* = 2.42 (*SD* = 0.56) on the AGI, *M* = 18.56 (*SD* = 13.84) on the DASS-21, and *M* = 38.07 (*SD* = 8.63) on the HGRC-PG.

Most participants (*n* = 506) indicated that they received support after the death of their loved one, implying that almost 1/4th, did not receive any support. Regarding social support, support from friends (*n* = 444) and family (*n* = 460) were the most common sources of support, as well as support from a partner (*n* = 164). Regarding formal support, support from a private psychologist/psychiatrist (*n* = 123) was mentioned the most. About 200 participants indicated that they wanted more support after the death of their loved one. More than half of these participants wanted help from a private psychologist or psychiatrist and about 1/3rd wanted support from a care provider within their university or college. Table 1 presents an overview of the received and desired support.

**Table 1:** Received and desired support.

	*n*	%
Did you receive support after the death of your loved one (formal/social)?		
Yes	506	76
No	160	24
Who did you receive support from after the death of your loved one? (*n* = 506)		
Friends	444	87.74
Family	460	90.9
Partner	164	32.41
Neighbors	36	7.11
General practitioner	56	11.06
Private psychologist/psychiatrist	123	24.3
Student counselor/teacher/lecturer	86	16.99
(Other) Care provider within the university/college	57	11.26
Social services	18	3.55
Support group	14	2.76
Selfhelp	23	4.5
Helpline	3	0.59
Online chat	8	1.58
Other		
Faith/pastor	3	0.59
Physiotherapist	1	0.19
Victim support/grief counselor	2	0.39
Psychiatric admission	1	0.19
	***n***	**%**
Would you have liked to receive more support?		
Yes	202	30.3
No	464	69.7
Who else would you have liked to receive support from? (*n* = 202)		
Friends	57	28.21
Family	55	27.22
Partner	18	8.91
Neighbors	/	/
General practitioner	17	8.41
Private psychologist/psychiatrist	111	54.95
Student counselor/teacher/lecturer	44	21.78
(Other) Care provider within the university/college	70	34.65
Social services	19	9.4
Support group	43	21.28
Selfhelp	8	3.96
Helpline	5	2.47
Online chat	7	3.46
Other	/	/

The first MANCOVA revealed a group effect regarding students who did or did not receive support after the bereavement: *F*(3, 660) = 33.371, *p* < 0.0005, Wilks’ Λ = 0.941, partial η^2^ = 0.059. There was a significant difference in grief (AGI; Andriessen et al., 2018) between participants who received (*M* = 2.46, *SD* = 0.57) and did not receive (*M* = 2.31, *SD* = 0.55) support after the death (*p* = 0.002, partial η^2^ = 0.014). There was also a difference regarding personal growth (HGRC-PG; Hogan et al., 2001) between participants who received (*M* = 39.09, *SD* = 8.08) and did not receive (*M* = 34.85, *SD* = 9.51) support (*p* < 0.000, partial η^2^ = 0.047). Thus, students who received support after the death of a loved one reported more grief and more personal growth than those who received no support. No significant difference was found between the two groups regarding mental health distress (DASS-21; de Beurs et al., 2001).

The second MANCOVA revealed a group effect regarding students who did or did not had wanted (more) support after the bereavement: *F*(3, 660) = 13.746, *p* < 0.0005, Wilks’ Λ = 0.868, partial η^2^ = 0.132. Participants who wanted more support had higher grief (AGI) scores (*M* = 2.71, *SD* = 0.53) than those who did not (*M* = 2.30, *SD* = 0.53) want more support (*p* < 0.000, η^2^ = 0.117). Participants who wanted more support scored also higher on mental health distress (*M* = 24.16, *SD* = 13.88) than those who did not (*M* = 16.13, *SD* = 13.11) wanted more support (*p* < 0.000, η^2^ = 0.073). There was no difference between the two groups regarding personal growth.

The questions regarding what students found important when receiving support and barriers to help-seeking were completed by 664 participants. Nearly all participants (*n* = 649) indicated that having their feelings recognized was very important in receiving support. In addition, a sense of safety (*n* = 637), help being offered actively (*n* = 500), and support that was available when they were ready (*n* = 644) were all considered important. Lastly, we found that participants found it important that support was provided as quickly as possible (*n* = 397) and that the person who provides the support has expertise and knowledge about coping with bereavement (*n* = 445).

More than half of the 664 participants (*n* = 359) indicated that they had experienced barriers that hindered them in seeking support from friends and/or family. The most common barriers were feeling like a burden (*n* = 258) and feeling uncomfortable when seeking support (*n* = 256). In addition, 207 participants indicated that they did not like to talk about their thoughts and feelings, and 183 participants felt that others needed more help than them. Almost half of the participants (*n* = 295) reported barriers in seeking professional support. Feeling uncomfortable was the most common barrier (*n* = 166). In addition, 122 participants did not know where or from whom to ask for help, and 114 participants did not feel a needed for support. Table 2 presents an overview of the reported barriers.

**Table 2:** Barriers in seeking social and formal support.

	*n*	%
Did anything hold you back from seeking support from family and/or friends? (*n* = 664)		
Yes	359	54.1
No	305	45.9
What held you back from seeking support from family and/or friends? (*n* = 359)		
I did not know where or who to turn to for support.	99	27.57
I felt uncomfortable seeking support.	256	71.3
I felt like a burden.	258	71.86
I had bad experiences seeking support.	67	18.66
I felt like I was being judged.	75	20.89
I felt guilty.	83	23.11
I did not feel I needed support.	86	23.95
I felt that others needed more support than I did.	183	50.97
I did not like talking about my thoughts and feelings.	207	57.66
I was limited by my financial resources.	22	6.12
Other		
I did not feel understood.	10	2.78
I did not want my friends to treat me differently/ my friends felt uncomfortable.	3	0.83
I wanted to deny what had happened.	1	0.27
I have a bad relationship with my family/they did not have time for me.	3	0.83
	***n***	**%**
Did anything hold you back from seeking support from a professional? (*n* = 664)		
Yes	295	44.4
No	369	55.6
What held you back from seeking support from a professional? (*n* = 295)		
I did not know where or who to turn to for support.	122	41.35
I felt uncomfortable seeking support.	166	56.27
I felt like a burden.	87	29.49
I had bad experiences seeking support.	56	18.98
I felt like I was being judged.	48	16.27
I felt guilty.	34	11.52
I did not feel I needed support.	114	38.64
I felt that others needed more support than I did.	104	35.25
I did not like talking about my thoughts and feelings.	109	36.94
I was limited by my financial resources.	70	23.72
Other		
I did not feel a connection with the professional.	1	0.33
I did not feel understood.	3	1.01
I was on the waiting list/the waiting lists were too long.	3	1.01
I did not have time for it.	1	0.33
I did not dare ask my parents for formal support/they did not understand/they thought it was taboo.	7	2.37

## Discussion

Most participants in our study received support from at least one person after the death of their loved one. Nonetheless, about 1 in 4 did not receive any support and about 30% reported that they wanted more support. Students who wanted more support reported more feelings of grief and mental health distress than those who felt that they had received enough support, indicating both vulnerabilities for mental health problems and unmet support needs in bereaved students.

We also found that students who received support after the death of a loved one reported more grief and more personal growth, but not significantly more or less mental health distress than participants who received no support. Talking about the death of their loved one can contribute to experiencing personal or posttraumatic growth, as found by Tedeschi and Calhoun (2004). Although talking about and processing the death can lead to positive outcomes over time, the process itself can temporarily increase the bereaved students’ feelings of grief due to them repeatedly having conversations with other people about their feelings. Abbott and Zakriski (2014) found that peer social support can prolong grief because of the repeated focus on and exchange of negative emotions, also referred to as co-rumination.

Participants indicated that they needed more professional support, as well as social support. Most participants in the studies by Dyregrov (2009) and Pitman et al. (2018) also indicated a need for professional help after experiencing a loss through suicide. As students are vulnerable for developing mental health problems after the death of a loved one, they may need support in navigating the grief process. Trained professionals can support students in their grieving process, and explore students’ strengths and how to use these in coping with negative feelings (Servaty-Seib and Taub, 2010).

When receiving support, our participants indicated a need for having their feelings acknowledged. They also expressed a need to feel safe when seeking support. Similarly, participants in the study by Summerhurst et al. (2017) reported the importance of safety, comfort, confidentiality, and not being judged when receiving formal support. These factors are crucial for students to be able to trust a clinician and express their emotions. Being able to share with others contributes to better coping skills in dealing with the death of a loved one (Cohen and Samp, 2018). Spence et al. (2016) found that young adults who use avoidant strategies (e.g., ignoring the problem) tend to use self-harm or substances as a destructive way of coping with problems. They also found that participants had the tendency to minimalize their problems. Bereaved students in a study by Czyz et al. (2013) did not seek professional help mainly because they felt that their problems were not serious enough. Importantly, a substantial number (*n* = 114) of participants in our study also identified this as a reason to not seek professional support, leaving them vulnerable for mental health deteriorations (Czyz et al., 2013).

Almost half of our participants experienced barriers in seeking formal and social support. This prevented some of these students from receiving the support they needed. Many students in our study expressed that they did not seek help or found it difficult to seek support because they felt like a burden to others. They also felt uncomfortable seeking support and did not like talking about their thoughts and feelings. In addition, a substantial group of bereaved students did not know where of who to turn to for social and/or formal support, which has also been reported in other studies (e.g., Andriessen et al., 2019; Hay et al., 2022).

Bereaved students expressed a wish that support was actively provided and as quickly as possible after the bereavement, a finding corroborated by the literature (Pitman et al., 2018), Nonetheless, some students may experience too much distress shortly after the death of their loved one to accept any support. Interestingly, Pitman et al. (2018) found that students who did not immediately looked for support found it difficult to seek support later because people expected that they had already recovered from their grief.

Besides the strengths of our study, some limitations need attention. The sample consisted mostly of female students, and our results may apply to students, but not to all bereaved emerging adults. Nonetheless, the study successfully recruited a substantial sample from across the country. We created the study survey before the COVID-19 pandemic, but data collection coincided with the onset of the pandemic. It is unknown if this has affected the results. In addition, the study relied on retrospective self-reporting, which may entail a recall bias, and the correlational findings preclude causal conclusions. Future research may use in-depth interviews to learn more about bereaved student’s experiences and needs in seeking and receiving support, or use prospective designs to investigate temporal relationships between study variables.

Our study sheds light on help-seeking behavior of students after the death of a loved one. The findings reveal the need for formal and social support for bereaved students to address their unmet grief and mental health concerns. Even though it can temporarily increase students’ feelings of grief, social and formal support can improve coping skills and positive mental health outcomes, including personal or posttraumatic growth (Tedeschi and Calhoun, 2004; Servaty-Seib and Taub, 2010).

Support should be offered shortly after the death, with support being accessible on an ongoing basis so that students can use it when they feel ready to accept it. It is important to not assume students have processed their grief because some time has passed or because they did not seem as close to the person who died. Bereaved students indicate a need for having their feelings acknowledged and feeling safe when receiving support, i.e., essential factors enabling students to share their feelings.

Formal support providers, such as healthcare professionals and university staff, should be trained in offering proactive support. This can lower multiple barriers, especially for students who are unsure where to ask for help and students who perceive their problems as not requiring any support, or do not want to bother others. It is important that social carers (e.g., parents, friends) receive advice on how to support a bereaved student in their grieving process.

## Data availability statement

The data supporting the conclusions of this article will be made available by the authors, upon reasonable request.

## Ethics statement

The studies was reviewed and approved by Social and Societal Ethics Committee of the KU Leuven—University of Leuven (G-2019 101762, March 19, 2020). The participants provided their written informed consent to participate in this study.

## Author contributions

ET, LC, and KA: conceptualization, methodology, and writing—reviewing and editing. ET: data collection, analysis, and writing—original draft preparation. LC and KA: supervision and project administration. All authors have read and agreed to the published version of the manuscript.

## Funding

KA was supported by a National Health and Medical Research Council Early Career Fellowship (1157796).

## Conflict of interest

The authors declare that the research was conducted in the absence of any commercial or financial relationships that could be construed as a potential conflict of interest.

## Publisher’s note

All claims expressed in this article are solely those of the authors and do not necessarily represent those of their affiliated organizations, or those of the publisher, the editors and the reviewers. Any product that may be evaluated in this article, or claim that may be made by its manufacturer, is not guaranteed or endorsed by the publisher.
